# Current status of primary, secondary and tertiary prevention of congenital cytomegalovirus disease: a call to action

**DOI:** 10.1097/QCO.0000000000001137

**Published:** 2025-08-12

**Authors:** Heather Bailey, Helen Payne, Hermione Lyall

**Affiliations:** a Institute for Global Health, University College London (UCL); b Section of Paediatric Infectious Disease, Imperial College London, London, UK; c Department of Paediatrics and Child Health, and Department of Immunology, Stellenbosch University, South Africa; d Department of Paediatric Infectious Diseases, Imperial College Healthcare NHS Trust, London, UK

**Keywords:** antivirals, congenital cytomegalovirus, deafness, neurodisability, prevention, screening

## Abstract

**Purpose of review:**

Globally, sequelae of congenital CMV (CCMV) impact an estimated 350 000 children born annually. In this review, we consider new evidence across primary, secondary and tertiary prevention approaches, and remaining evidence gaps.

**Recent findings:**

Education on hygiene precautions can reduce risk of primary CMV acquisition in pregnancy, and may have a role in some settings in reducing CCMV cases resulting from nonprimary infection, but public and health worker knowledge and awareness remains low. Evidence that valaciclovir treatment can reduce CMV vertical transmission has led to renewed interest in antenatal CMV screening in some high-income countries over recent years, although there is a lack of recommendation in most guidelines and significant evidence gaps remain. Newborn CCMV screening has been adopted in some states/provinces in Canada/USA, with first results recently published. Newborn prognostic scoring systems are evolving, with potential for more effective targeting of newborn treatment and tertiary prevention of CCMV disease.

**Summary:**

We make suggestions for clinical practice and research, particularly to address evidence gaps around: safety and effectiveness of antenatal CMV screening and antiviral prophylaxis; findings relating to detection of nonprimary infection in pregnancy; new prognostic neonatal scoring systems; and learning from follow-up of children born into state-wide universal CMV screening programmes.

## INTRODUCTION

**FIGURE 1:**
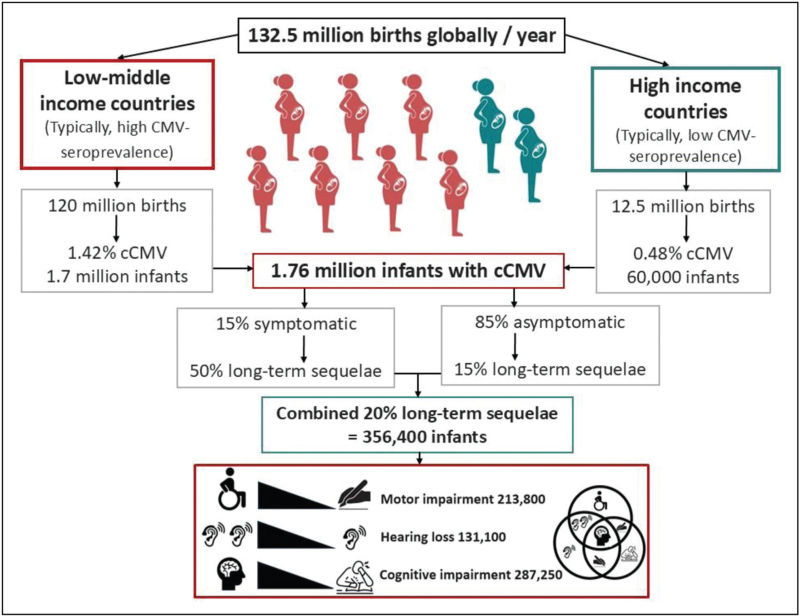
Global prevalence of congenital cytomegalovirus and sequelae. This figure has used global birth data [4] and two studies that reported frequencies of long-term sequelae [2,3] to estimate numbers of infants severely affected by CCMV annually. These studies utilized universal screening for CCMV from 2007 and 2017, although the more recent study required parental consent for retrospective testing of dried blood spots and then data linkage to outcomes, which may have introduced an element of bias in reported prevalence of sequelae. The Venn diagram is a visual representative of potential overlap between domains of sequelae but not based on absolute numbers as follow-up data is limited. Infants with symptomatic disease with long-term sequelae more frequently have a combination of hearing loss, motor and cognitive impairment.

Globally, congenital cytomegalovirus (CCMV) is by far the commonest congenital infection, although arguably, the least well recognized by the public and healthcare professionals (HCP), and least well researched considering its frequency and consequences. A meta-analysis of neonatal screening studies found a global CCMV birth prevalence of 0.67% [95% confidence interval (CI) 0.54–0.83%], and three-fold higher in low-income and middle-income countries (LMICs) than high-income countries (HIC) [1]. This reflects the importance of nonprimary maternal CMV infection in the epidemiology of CCMV, with prepregnancy CMV-seroprevalence of more than 90 and 50–60% in LMICs and HICs, respectively [1]. Worldwide, there are an estimated 1 760 000 infants born annually with CCMV, 356 400 of whom have CMV-related long-term sequelae [2–4] (Fig. 1). Routine neonatal screening for CCMV remains rare, with state-wide screening only underway in: Ontario, New York State and Minnesota [5^▪▪^,^6^^▪▪^]. 

**Box 1:**
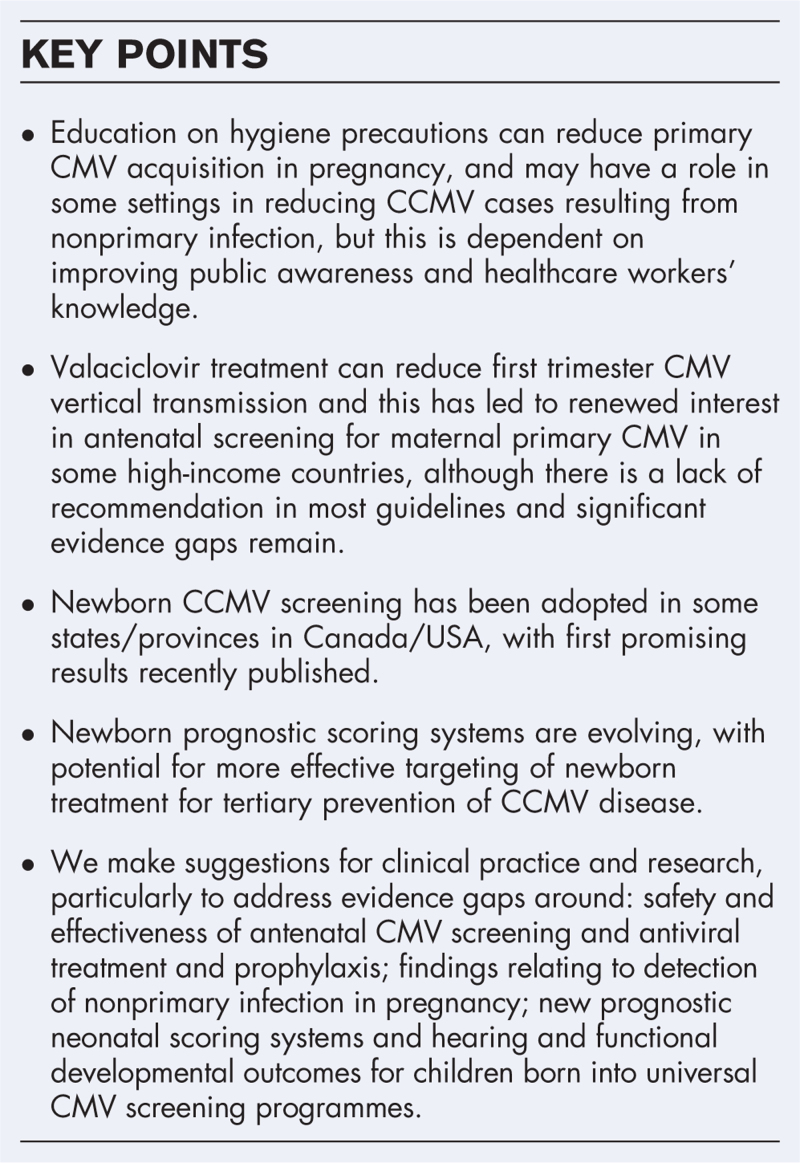
no caption available

In-utero transmission of CMV, usually following asymptomatic maternal infection, causes a spectrum of newborn effects, from severe life-limiting disease to no apparent impact, although most infants are at the less severe end of the spectrum. Following primary infection, transplacental CMV transmission in early pregnancy leads to significant sequelae including sensorineural hearing loss (SNHL), and brain injury with severe neurodevelopmental consequences in up to a fifth of infants [7]. This has been described as the embryopathy of first trimester CCMV, with structural and inflammatory tissue damage to foetal brain, cochleae, and vestibular apparatus [8,9]. In contrast, foetal infection in the second or third trimester appears much less likely to be associated with long-term consequences, although further studies with longer term follow-up are needed to fully understand the long-term impacts of cCMV. CMV-seronegative women with a short inter-pregnancy interval are at particular risk for a first trimester primary CMV infection in a subsequent pregnancy, with a French study indicating that risk peaked at 7% with an inter-pregnancy interval of 1–2 years [10].

Transmission rates following maternal nonprimary infection are difficult to determine due to challenges establishing CMV reinfection or reactivation during pregnancy but are estimated at 3.5% or lower [11]. CCMV birth prevalence overall increases with higher HIV prevalence [1]. Although vertical transmission rates are higher among mothers with primary seroconversion, worldwide, many more infants with CCMV are born to women who are already seropositive, and most are not diagnosed, with no access to treatment [12].

Initial categorization of infants with CCMV as clinically ‘symptomatic’ or ‘asymptomatic’ at birth predated newborn hearing screening and brain imaging; a significant proportion of infants who examine normally at birth may have SNHL and/or abnormal findings on brain MRI. Clinical follow-up should continue until school age to identify late-onset and progressive SNHL, and other neurodevelopmental sequelae [13^▪▪^], but studies with this length of follow-up are limited [14].

**FIGURE 2:**
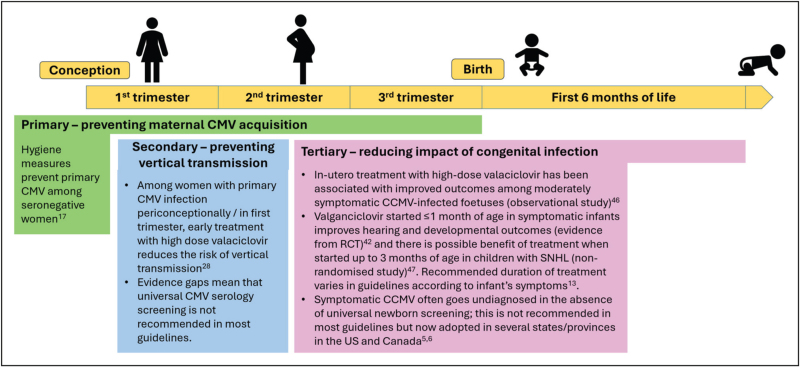
Key points for primary, secondary and tertiary prevention of congenital cytomegalovirus disease. Superscript numbers indicate references in the main text.

Economic costs of CCMV are high, including lifelong support for children with cerebral palsy and epilepsy, as well as cochlear implantation for profound SNHL, although costs are more difficult to assess for those less severely affected. Recent studies suggest that universal neonatal screening is more cost-effective than targeted testing to reduce CCMV-related sequelae [15,16]. Although the foetal effects of CCMV can be mitigated by treatment after birth, they cannot be cured like congenital syphilis, or fully suppressed like HIV. As for these other prevalent congenital infections, the best way to protect infants from CCMV is to prevent vertical transmission. Here, we review recent evidence across primary, secondary and tertiary prevention of CCMV disease (Fig. 2).

## PRIMARY PREVENTION: PREVENTION OF ACQUISITION OF CYTOMEGALOVIRUS IN PREGNANCY

Provision of hygiene information for CMV-seronegative pregnant women can reduce the proportion seroconverting antenatally, with high acceptability, as demonstrated in a controlled intervention study [17]. However, an Italian study, which aimed to assess a similar intervention in CMV-seropositive women did not proceed due to lower rates of CCMV than anticipated [18]. Authors used several methods to attempt to distinguish between reinfection or reactivation of maternal CMV where CCMV had occurred. Where available, genotype-specific IgG showed that only one of seven women had a potential reinfection with a new CMV strain. In addition, lack of association between CCMV and maternal contact with young children as well as increased risk of transmission among women with comorbidities, particularly diabetes, were taken as indirect evidence that in this cohort, most transmissions were due to reactivation rather than reinfection [18]. In contrast, reinfection with new CMV strains have been responsible for vertical transmission in other studies (e.g. in Brazil [19]) with reinfection identified in 2.7% of CMV-seropositive pregnant women in a recent study in Canada [20^▪^]. Thus, the potential for hygiene recommendations to prevent CCMV among seropositive women is likely to vary by setting. Many HCPs remain inadequately informed to impart CMV hygiene advice, and there is a lack of stakeholder advocacy to integrate CMV knowledge into routine antenatal care [21].

CMV vaccine studies have been underway for over 50  years, and seven candidate vaccines are currently under trial [22]. Results of a CMV mRNA phase III randomised, placebo-controlled vaccine trial in CMV-seronegative women of child-bearing age, exposed to young children, are awaited [23]. In future, a potential CMV vaccine could be included in routine childhood immunizations, offered to adolescents, or to CMV-seronegative women planning pregnancy [24]. However, protection of infants of CMV-seropositive women requires significant progress to be made in understanding how to counteract the multiple ways in which CMV evades the host immune response [25]. An in-vitro placental immunology study in seropositive women found that CMV-specific tissue-resident CD8+ T cells in decidual tissues can exert cytotoxic effector functions, and provide antiviral protection upon CMV-reinfection, which could inform future vaccine development approaches [26].

## SECONDARY PREVENTION: PREVENTION OF VERTICAL TRANSMISSION OF CMV

It is 5 years since publication of a randomized controlled trial showing that high-dose valaciclovir reduces CMV vertical transmission following primary CMV infection in the first trimester [27]. An individual patient data meta-analysis including this trial and two quasi-randomized studies showed a similar reduction in risk of vertical transmission with valaciclovir following periconception primary infection [adjusted odds ratio (aOR) 0.34, 95% CI 0.18–0.61 for periconception and aOR 0.35, 95% CI 0.16–0.76 for first-trimester infection], with higher rates of vertical transmission when valaciclovir was initiated at a later gestational week [28^▪▪^].

The efficacy of valaciclovir has prompted off-label use in pregnant women identified with primary CMV [29]. There is renewed interest in first trimester serological screening in HICs [13^▪▪^], and this is increasingly being adopted, despite lack of recommendation in most guidelines. For example, in Italy [30] and in Germany, where up to 40% of pregnant women are tested at their own expense [31]. In Spain, a recent first trimester pilot study in a population with a CMV seroprevalence of 70.6%, found that 0.14% of 2777 women screened had primary CMV infection, and vertical transmission occurred in one of four treated pregnancies, after valaciclovir was initiated late [32^▪▪^]. A French modelling study demonstrated that universal antenatal CMV screening with valaciclovir as prophylaxis would be cost-saving versus current practice, where only 25–50% of women are screened [33].

However, a recent opinion piece argued that criteria for a universal antenatal CMV screening have not yet been met, due to a lack of evidence of the safety and efficacy of screening programmes based on universal first-trimester serological screening followed by valaciclovir as secondary prevention [34]. There are also gaps in understanding of the epidemiology of CMV and acceptability of the screening pathway in different settings. For example, a recent Australian qualitative study highlighted the psychological impact of a suspected CMV infection in pregnancy, intensified by lack of CMV knowledge in family support networks and HCPs [35]. Concerns have also been raised that positive screening tests may result in termination of pregnancies with a good chance of a healthy outcome [36]. Importantly, criteria for the introduction of population screening state that other options such as primary prevention strategies, should be implemented as a prerequisite [37] and yet awareness of CMV and preventive interventions, among HCP and pregnant women remains low [21].

Importantly, serological screening cannot identify nonprimary maternal CMV infections, which account for a significant proportion of CCMV cases, even in low-seroprevalence HICs (e.g. 50% of CCMV cases in France [38]). Recent Dutch studies have used DNA sequencing data generated by first trimester non-invasive prenatal testing (NIPT) for foetal aneuploidy screening to identify maternal blood samples containing CMV cell-free DNA fragments [39^▪▪^,40^▪^]. There is evidence that these fragments could arise as a result of maternal infection and infection of the placenta and may identify both primary and nonprimary CMV infections in the first trimester [39^▪▪^,40^▪^]. Incorporating CMV screening into an existing antenatal NIPT screening programme has potential logistical and cost benefits, but prospective clinical studies are required to link these findings with clinical outcomes.

In parallel with changes in use of CMV testing in pregnancy, there have been changes in the epidemiology of children diagnosed with CCMV in Europe, with an increasing proportion diagnosed, including some treated, *in utero*, and often without clinically apparent symptoms at birth [41]. The implications of diagnosis and treatment *in utero* should be explored by routine collation of data, alongside outcomes for children identified with CCMV after birth.

## TERTIARY PREVENTION: LIMITATION OF SEVERE CONSEQUENCES OF CONGENITAL CYTOMEGALOVIRUS DISEASE

Two randomized controlled trials of ganciclovir/valganciclovir demonstrated a significant reduction in progressive and late-onset hearing loss, and improvement in developmental outcomes in symptomatic infants born more than 32 weeks gestation, birthweight greater than 1.8 kg, initiating therapy at less than 1 month of age [42]. Treating early makes biological sense, as treatment is more likely to be beneficial during immunological immaturity of early infancy when there is reduced ability to control CMV, and indeed, makes the case for treatment *in utero* with antenatal diagnosis [43–46]. In a more recent nonrandomised study in the Netherlands, children born at term (≥37 weeks) with normal birthweight, unilateral or bilateral hearing loss (≥21 dB), and diagnosed with cCMV through targeted screening incorporated into the Newborn Hearing Screening Programme without prior clinical suspicion, had the option of initiating valganciclovir up to 13 weeks of age. Whether to initiate treatment was determined by parental choice; children receiving 6 weeks of valganciclovir had reduced subsequent hearing loss up to 20 months of age, as compared with a control group (those who chose nontreatment and historical controls) [47]. However, a small randomised study of 6 weeks valganciclovir versus placebo for children diagnosed with CCMV and SNHL aged 1 month to 3 years of age (up to their fourth birthday) did not demonstrate improved hearing outcomes 6 months later [48]. Small and heterogeneous study populations, complexities of audiological testing, and short and incomplete follow-up, are among the methodological issues that complicate interpretation of these and many CCMV studies.

Early treatment initiation depends on early diagnosis, and screening studies have shown that even infants symptomatic at birth can be clinically missed [6^▪▪^]. Barriers to universal screening include: uncertainty regarding whether screening tests will identify those most at risk of long-term neurodevelopmental and hearing problems; whether asymptomatic infants require treatment or not; and whether the investigation of asymptomatic infants leads to undue cost and parental anxiety [49]. In Ontario, these concerns have been closely examined, and subsequent to the pilot study, universal neonatal screening will continue [6^▪▪^].

In the absence of universal screening, targeted CMV testing of high-risk groups less than 3 weeks of age is an alternative approach including: premature infants; HIV-exposed; infants with growth restriction; or infants who have failed new-born hearing screening. However, this relies upon HCPs’ awareness and local policies, and there is considerable disparity in service delivery [50].

Although there is evidence for early treatment in symptomatic infants, this is lacking for those considered ‘asymptomatic’, a proportion of whom may have hearing loss and or abnormal findings on MRI [51], although an observational study has suggested treatment initiated for infants with milder hearing loss at diagnosis had high likelihood of hearing improvement [52]. Neonatal prognostic scores, constructed from clinical, biochemical and radiological features are being developed to predict long-term outcomes across the spectrum of CCMV, to identify with increased precision which infants will benefit from treatment. In a cohort of 227 children, absence of SNHL at birth, with normal cerebral ultrasound and platelet count, was predictive of no risk of neurologic sequelae, and a low risk of delayed unilateral SNHL up to 2 years of age [53^▪^]. A prognostic score focusing upon identifying infants with progressive CMV disease is also being developed from a prospective cohort of 1500 children with follow-up to school age in the European CCMVNET registry. Prospective follow-up of infants born into recent state screening systems will provide further insight in predicting consequences of mildly affected or asymptomatic infants [5^▪▪^,^6^^▪▪^]. Emerging evidence for antenatal and neonatal biomarkers for CCMV long-term sequelae may be useful additions to baseline clinical findings include immunomodulatory proteins [54], and plasma and cell-associated CMV reservoirs [55], and a 13-gene signature predictive of late-onset hearing loss [56]. Recent additions to this field include magnitude of amniotic CMV viral load [57] or reduced or absent CMV-specific neonatal immune responses associated with developmental delay, and high levels of exhausted CD8 T cells in children with progressive SNHL [43]. The validated CCMV-specific brain MRI score correlates neonatal imaging findings in symptomatic infants with developmental outcomes at median 3–4 years of age, also adding to the predictive neonatal battery of investigations [58^▪▪^]. However, for predictive approaches to be reliable, systematic measures of functional developmental outcomes should also be applied, and described alongside environmental context and other developmental support [59].

Ganciclovir/valganciclovir remains unlicensed for treatment of cCMV in neonates. Evidence-based rationale for treatment is essential, particularly in LMICs where rates of CCMV are higher [60] and side-effect monitoring challenging. Neutropenia and transaminitis occur in up to 46% of treated term and preterm infants [61], and animal data implies potential risk of teratogenicity and carcinogenesis [13^▪▪^]. Important treatment issues, which remain to be addressed include: optimal duration of treatment; dosing for premature infants with CCMV; best treatment for infants with HIV and CCMV; and how to improve treatment outcomes while minimizing side effects. Alternative approaches to be evaluated could include, valganciclovir to suppress CMV, followed by antivirals with less toxicity to maintain suppression.

The value of psychological support for families in managing an unexpected and life-changing newborn diagnosis, and the challenges that lie ahead managing a child with disability, should not be overlooked [13^▪▪^]. A holistic approach informed by the lived experience of families affected by CCMV, and including integrated clinical psychology support, will ultimately improve clinical, developmental and socio-emotional outcomes for the child and family.

## CONCLUSION

Despite recent progress, many questions remain unanswered, including better understanding of the immunology and virology of CMV vertical transmission, and optimal approaches for secondary and tertiary prevention of CCMV-related sequelae (Table 1). However, there are key clinical interventions that can be implemented now, which reflect a shift towards prevention of CMV infection, alongside addressing treatment. Indisputably, the immediate focus should be upon public and HCP education (including awareness of hygiene-based prevention measures), alongside developing and strengthening equitable and evidence-based pathways for antenatal and postnatal management, even in the absence of universal screening. Advocacy from the CCMV community has built international recognition of the global impact of CCMV for children and families, health and social care services. It is now time for the clinical and academic community to step-up and collaboratively apply the evidence we have for pragmatic clinical practice while on-going and new scientific studies address the gaps in understanding.

**Table 1:** Clinical practice and research recommendations

Clinical practice
Education of public and healthcare workers on CCMV: Primary prevention hygiene measures to reduce the risk of CMV acquisition during pregnancy Features of neonatal presentation, to improve diagnosis of symptomatic infection.
Establishing and evaluating management and treatment pathways for antenatally diagnosed CMV, for prophylaxis of VT and foetal treatment, to ensure equitable and evidence-based options and family support.
Use of predictive neonatal scoring approaches: cCMV-specific brain MRI, to assess severity and need for treatment Normal cranial ultrasound scan, normal hearing and platelets at diagnosis identifies low-risk infants who may not require intensive follow-up.
Research priorities
Epidemiological research to investigate the role of first trimester screening and antenatal antiviral prophylaxis in reducing CCMV disease burden
Clinical, implementation and social science research to evaluate the effectiveness and safety of antenatal CMV screening and antiviral prophylaxis in reducing CCMV, and acceptability of the antenatal screening pathway in real-world settings.
Health economic assessments of universal screening of first trimester primary maternal CMV infection and antiviral prophylaxis of vertical transmission; screening and treating neonatal CCMV.
Further research on the detection of first trimester maternal blood CMV DNA fragments to diagnose nonprimary as well as primary maternal CMV infection, and how this links with vertical transmission and clinical outcomes.
Development and evaluation of prognostic value of new neonatal scoring systems for CCMV.
Immunology, virology, transcriptomic/proteomic profiling studies to better identify biomarkers for severity of CCMV
Long-term follow-up of modern cohorts (LMIC and HIC) of children with CCMV to at least 5–6 years of age, to ascertain true burden of disease, across the spectrum of symptomatology.
Randomized controlled trials of new/different antiviral combinations for CMV suppression, both antenatally and postnatally.

CCMV, congenital cytomegalovirus; HIC, high-income countries; LMICs, low-income and middle-income countries; VT, vertical transmission.

## Acknowledgements

*None*.

### Financial support and sponsorship

*None.*

### Conflicts of interest

*H.B. is a co-investigator of the European Pregnancy and Paediatric Infections Cohort Collaboration (EPPICC), which has received funding from ViiV Healthcare and Merck Sharp & Dohme via Penta Foundation and a member of the Advisory Group for the UK National Screening Committee review of evidence on CMV screening.*

## REFERENCES AND RECOMMENDED READING

Papers of particular interest, published within the annual period of review, have been highlighted as:

▪ of special interest
▪▪ of outstanding interest
